# Supplementary material for: Driving Factors Influencing Soil Microbial Community Succession of Coal Mining Subsidence Areas during Natural Recovery in Inner Mongolia Grasslands
Source: Microorganisms. 2023 Dec 31;12(1):87. doi: 10.3390/microorganisms12010087 (PMC10818900; doi:10.3390/microorganisms12010087)
Supplement: Supplementary file 1 [file microorganisms-12-00087-s001.zip › microorganisms-2781964-supplementary.pdf]

Table S1. Main Plants in the Study Area.

Family name	Lifestyle	Latin scientific name
Plantaginaceae Juss.	Biennial or perennial herb	**Plantago asiatica** L.
Euphorbiaceae Juss.	Annual herb	**Euphorbia humifusa** Willd.
	Annual herb	**Euphorbia thymifolia** L.
Fabaceae Lindl.	Perennial herb	**Astragalus laxmannii** Jacq.
	Perennial herb	**Medicago sativa** L.
	Biennial herb	**Melilotus officinalis** (L.) Pall.
	Perennial herb	**Astragalus scaberrimus** Bunge
	Perennial herb	**Oxytropis coerulea** (Pall.) DC.
Poaceae Barnhart.	Perennial herb	**Leymus chinensis** (Trin.) Tzvel.
Apocynaceae Juss.	Grassy or subshrub-like vines	**Cynanchum thesioides** (Freyn) K. Schum.
Asteraceae Bercht. & J. Presl.	Perennial herb	**Inula britannica** Linnaeus
	Perennial herb	**Taraxacum mongolicum** Hand.-Mazz.
	Perennial herb	**Takhtajaniantha mongolica** (Maxim.) Zaika, Sukhor. & N. Kilian
	Perennial herb	**Artemisia mongolica** (Fisch. ex Bess.) Nakai
	Perennial herb	**Artemisia integrifolia** L.
	Annual herb	**Parthenium hysterophorus** L.
	One or two years of	**Artemisia sieversiana** Ehrhart ex Willd.

	herbs	
	Perennial herb	**Artemisia lavandulifolia** Candolle
	Perennial herb or one/two year herb	**Artemisia scoparia** Waldst. et Kit.
	Perennial herb	**Crepis crocea** (Lam.) Babcock
	Perennial herb	**Leibnitzia anandria** (Linnaeus) Turczaninow
	Perennial herb	**Artemisia desertorum** Spreng.
	Perennial herb	**Saussurea hieracioides** Hook. f.
	Perennial herb	**Ixeris chinensis** (Thunb.) Nakai
	Perennial herb	**Cirsium arvense** var. **integrifolium** C. Wimm. et Grabowski
Polygonaceae Juss.	Annual herb	**Rumex maritimus** L.
Orobanchaceae Vent	Biennial or perennial parasitic herb	**Orobanche pycnostachya** Hance
Geraniaceae Juss.	Perennial herb	**Geranium albiflorum** Ledeb.
	Perennial herb	**Geranium sibiricum** L.
Rosaceae Juss.	One or two years of herbs	**Potentilla supina** L.
	Perennial herbaceous or subshrub	**Sibbaldianthe bifurca** (L.) Kurtto & T. Erikss.
Apiaceae Lindl.	Perennial	**Bupleurum smithii** Wolff

	herb	
Brassicaceae	One or two years of herbs	**Lepidium apetalum** Willd.
Amaranthaceae Juss.	Annual herb	**Chenopodium bryoniifolium** Bunge
	Annual herb	**Chenopodium album** L.
	Annual herb	**Bassia scoparia** (L.) A.J.Scott
	Annual herb	**Kali collinum** (Pall.) Akhani & Roalson
	Annual herb	**Oxybasis glauca** (L.) S. Fuentes, Uotila & Borsch
Convolvulaceae Juss.	Perennial herb	**Convolvulus arvensis** L.
Boraginaceae Juss.	Perennial herb	**Trigonotis radicans** (Turcz.) Stev.
